# Supplementary material for: Evaluation of Quality Parameters and Functional Activity of Ottobratica Extra Virgin Olive Oil Enriched with Zingiber officinale (Ginger) by Two Different Enrichment Processes during One-Year Storage
Source: Foods. 2023 Oct 18;12(20):3822. doi: 10.3390/foods12203822 (PMC10606614; doi:10.3390/foods12203822)
Supplement: Supplementary file 1 [file foods-12-03822-s001.zip › Supplementary (figure+caption).pdf]

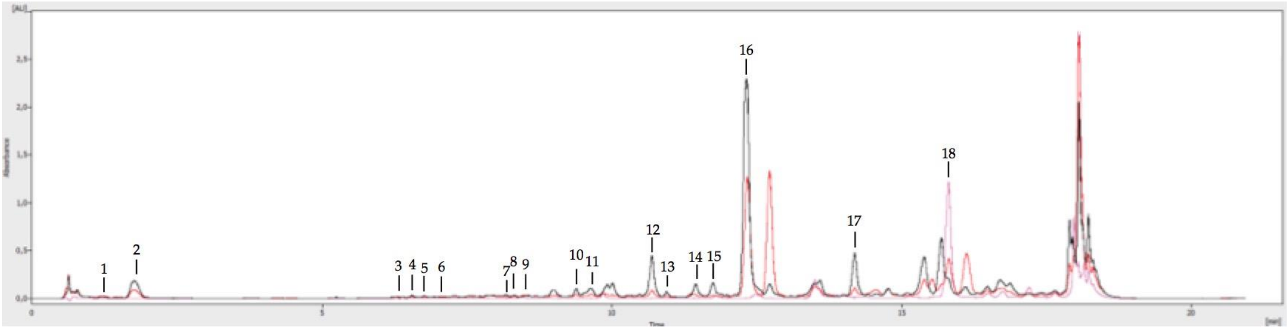

Figure S1. Chromatogram of ginger extract. 1: Gallic acid; 2: 3, 4-dihydroxybenzoic acid; 3: Chlorogenic acid; 4: Vanillic acid; 5: Caffeic acid; 6: Syringic acid; 7: *p*-Coumaric acid; 8: Ferulic acid; 9: Luteolin-7-*O*-glucoside; 10: Rutin; 11: Quercetin; 12: Apigenin; 13: Naringenin; 14: Kaempferol; 15: Isoramnetin; 16: 6-gingerol; 17: 6-shogaol; 18: Apigenin 7-*O*-glucoside.

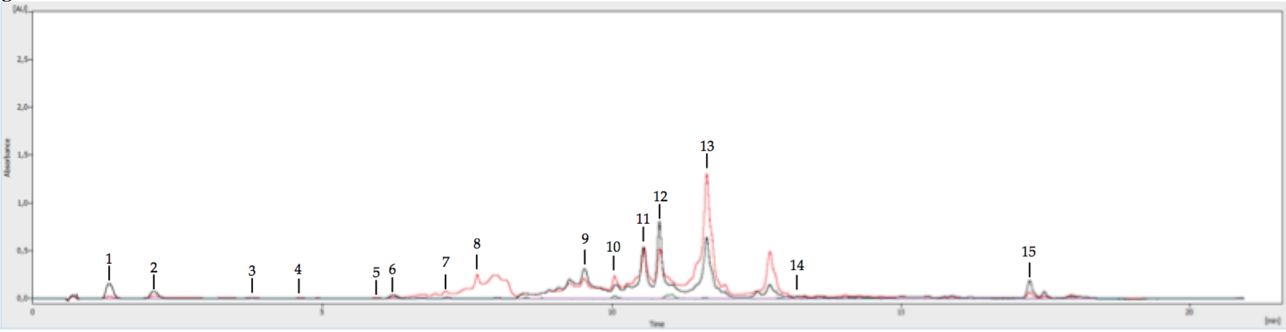

Figure S2. Chromatogram of EVOO (extra virgin olive oil). 1: Hydroxytyrosol; 2: Tyrosol; 3: Chlorogenic acid; 4: Vanillic acid; 5: Homovanillic acid; 6: *p*-Coumaric acid; 7: Luteolin-7-*O*-glucoside; 8: Quercetin 3,4′-diglucoside; 9: Oleuropein 10: Cinnamic acid; 11: Quercetin; 12: Pinoresinol; 13: Apigenin; 14: Isoramentin 3-*O*-gluoside; 15: Apigenin 7-*O*-glucoside.

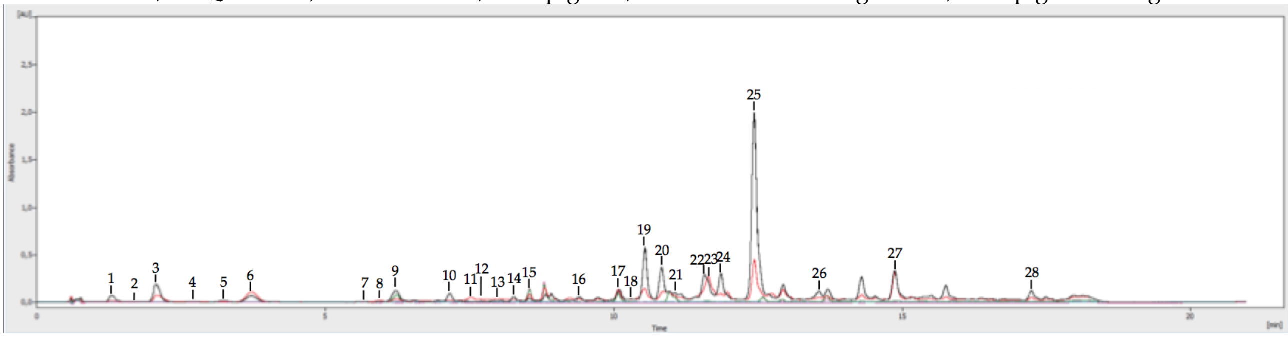

Figure S3. Chromatogram of GM (ginger flavoured olive oil by malaxation). 1: Hydroxytyrosol; 2: 3, 4-dihydroxybenzoic acid; 3: Tyrosol; 4: 4-hydroxyphenyl acetate; 5: Chlorogenic acid; 6: Vanillic acid; 7: Caffeic acid; 8: Homovanillic acid; 9: Vanillin; 10: *p*-Coumaric acid; 11: Quercetin 3,4′-diglucoside; 12: Ferulic acid; 13: Rutin; 14: *o*-Coumaric acid; 15: Luteolin-7-*O*-glucoside; 16: Oleuropein 17: Cinnamic acid; 18: Luteolin; 19: Quercetin; 20: Pinoresinol; 21: Naringenin; 22: Kaempferol; 23: Apigenin; 24: Isoramnetin; 25: 6-gingerol; 26: Isoramentin 3-*O*-gluoside; 27: 6-shogaol; 28: Apigenin 7-*O*-glucoside.

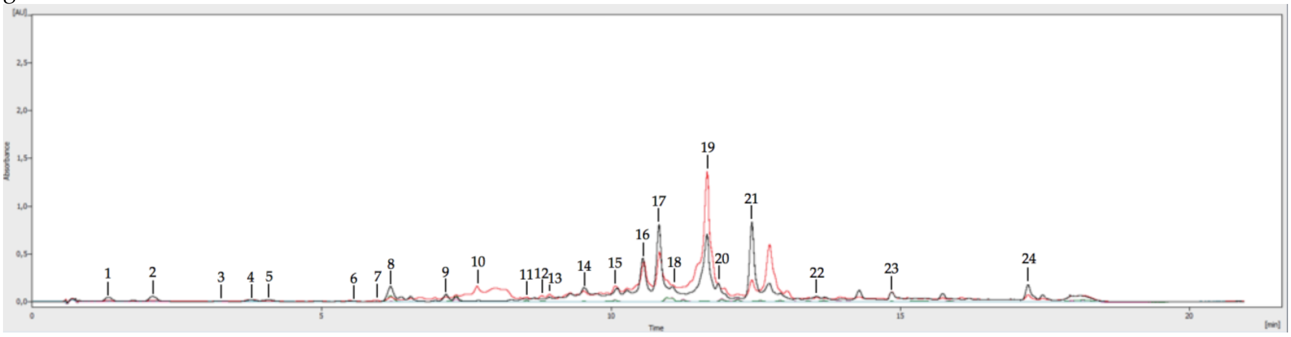

Figure S4. Chromatogram of GI (ginger flavoured olive oil by infusion). 1: Hydroxytyrosol; 2: Tyrosol; 3: 4-hydroxyphenyl acetate; 4: Chlorogenic acid; 5: Vanillic acid; 6: Caffeic acid; 7: Homovanillic acid; 8: Vanillin; 9: *p*-Coumaric acid; 10: Quercetin 3,4′-diglucoside; 11: Ferulic acid; 12: Rutin; 13: Luteolin-7-*O*-glucoside; 14: Oleuropein 15: Cinnamic acid; 16: Quercetin; 17: Pinoresinol; 18: Kaempferol; 19: Apigenin; 20: Isoramnetin; 21: 6-gingerol; 22: Isoramentin 3-*O*-gluoside; 23: 6-shogaol; 24: Apigenin 7-*O*-glucoside.
